# Hospital Meetings

**Published:** 1904-04-09

**Authors:** 


### HOSPITAL MEETINGS.

### ST. PETER'S HOSPITAL.

THE thirty-sixth annual meeting of Governors of St. Peter's Hospital for Stone and Urinary Diseases, Covent Garden, was held on Tuesday, March 29th, the chair being taken by Mr. Edwin Fox, one of the trustees. There was a good attendance.

The Chairman, in moving the adoption of the report and accounts for 1903, alluded to the absence, owing to family bereavement, of the treasurer, Mr. F. A. Bevan, who it had been hoped would have been able to preside over the annual meeting. On behalf of the governors he desired to express their sympathy with Mr. Bevan and the hope that he would be able, on a future occasion, to take his customary place in the chair. The report, the chairman continued, showed a very material improvement, not only in the work accomplished, but also in the finances of the hospital. It would be observed that 556 patients had been admitted during the year, nearly all of whom were either cured or relieved, while the number of deaths was only 28. The number admitted showed an increase of 75 on that of the previous year, so that it would be evident that the resources of the hospital had been strained to their utmost. Indeed, the want of additional accommodation was felt by the committee to be so urgent that they had asked Mr. Frederick W. Hunt, F.R.I.B.A., for his advice as to the best means for enlarging the capacity of the building, and Mr. Hunt, having kindly given his services as honorary consulting architect, plans for adding perhaps a dozen or more beds were now under consideration. For this, of course, funds were required, and, having a small balance in hand, the committee proposed to take the initial steps, regarding this sum as a nucleus, to which it was hoped additions would be made by the subscribers and other friends. The sum of £700, received in legacies since the beginning of the current year, had been allocated to this purpose, and he invited the supporters of the hospital to rally round the committee, and enable them to carry out this most necessary work. As a proof of the increase of work done in the hospital, he might mention that it had been found necessary to engage a second resident medical officer; a junior house surgeon had therefore been appointed. A special feature of the work of this hospital was the appreciation shown by medical and surgical practitioners of the opportunities for attendance at operations; there had been an increase of 140 over the numbers for the previous year the attendances being 1,141 during 1903. It was of the very highest importance that knowledge concerning the special branch of surgery practised at this hospital should be disseminated throughout the profession, and the increase in numbers to which he had alluded was therefore very encouraging. Turning to the question of the accounts, the chairman said he thought the governors might consider themselves fortunate, in these days of depression, since the contributions, both from patients and subscribers, showed an increase, the figures being some £400 in excess of those for the previous year. The annual subscriptions amounted to £1,391, while patients' payments reached the sum of £2,490, of which £2,398 was made up of voluntary contributions from out-patients. The committee desired most cordially to thank the governors and their friends for the very generous way in which they had responded to their appeal for funds, and at the same time to acknowledge the zeal with which the secretary and staff had worked to bring about such a satisfactory result. He hoped, however, that the help already given was only an augury of further assistance during the coming year. The task of enlarging the capacity of the hospital would necessarily be an expensive one, and it was essential, in view of the facts he had set forth, that past efforts should be redoubled. He concluded with a sympathetic reference to the death of Mr. F. Crockford, for twenty years an active member of the committee of management and a zealous friend of the hospital.

Mr. T. Christy, in seconding the motion, dwelt on the increased work and responsibility thrown upon the surgical and nursing staff, consequent upon the larger number of patients received during the year under review. He added that it was a matter for regret that the grant received from King Edward's Hospital Fund amounted only to £100; he thought they deserved a larger grant.

Mr. F. Swinford Edwards, F.R.C.S., in commenting upon the medical report, remarked on the low death-rate, viz., 5 per cent., of nearly 500 major operations performed during the year. He thought this result extremely good, when it was remembered that the cases admitted were of the most serious kind met with in surgical work. The surgeons heartily welcomed the news that the hospital was to be enlarged, and he urged that, in planning the alterations, room should be provided for a pathological department, for lack of which the staff was seriously hampered. The chemical, microscopical, and bacteriological examination of pathological material in a hospital of this kind was an absolute necessity, both for the sake of the patients and the staff, while the provision of such a department would greatly add to the value of the hospital as a teaching institution.

The usual votes of thanks concluded the meeting.

### ROYAL SEA-BATHING HOSPITAL, MARGATE.

The annual Court of Governors was held at the London offices of this hospital, 13 Charing Cross, on March 30th. The chair was taken by Sir Henry F. Lennard, who in his opening speech alluded to the loss sustained by the hospital through the death of the Duke of Cambridge, one of their vice-patrons, and also through the death of Mr. C. A. Swinburne, a director and vice-president of the hospital, and one of its most generous supporters. Besides being an annual subscriber Mr. Swinburne had given over £25,000 to the hospital during his life-time. Sir Henry Lennard then moved a resolution stating the deep loss sustained by the hospital through the death of Mr. Swinburne, and his resolution was seconded by Colonel Elliot, who bore testimony to the keen personal interest in the affairs of the hospital that Mr. Swinburne had always taken.

The Chairman then moved the adoption of the report, and said it was a satisfactory one on the whole. A second donation of £500, conditionally offered by "Well-Wisher," had been received in June last, the conditions having been fulfilled. This donation was for the purpose of recouping the invested funds upon which they had been compelled to entrench. They were glad to note a further increase in the Hodgson Five-Shillings Fund. The expenditure last year had been heavy, partly owing to an increase in the number of patients under treatment. The enlargement and refitting of the kitchen had cost £1,103, but this outlay was necessary, as the old kitchen was quite inadequate to meet the demands made upon it. Since its completion a more liberal dietary had been sanctioned. The demand for beds had again increased, the average daily number of patients in residence in 1903 having been 138 against 123 in 1902 and 119 in 1901. They had been told when they were compelled to raise the rate of patients' payments that their numbers would fall off, but this prophecy had been disproved. A child from the Sandringham Estate had been discharged cured, and in connection with this case the King had graciously contributed a donation of 30 guineas. After much careful consideration it had been decided to abolish the dual control by a superintendent and a matron at the hospital, and to unite the two offices in a matron-superintendent, who would be assisted by a sub-matron and a house-steward. The clerical work would be transferred to the London office. Lady Decies had collected a sum of £100 towards the £250 required to provide a complete *x*-rays department for the treatment of lupus.

Mr. Bertram Thornton seconded the resolution and referred with satisfaction to the more suitable nature of the cases sent to the hospital. Formerly cases in the last stages of tuberculosis were frequently sent, who were past all hopes of recovery, and only blocked up the beds and poisoned the hospital.

The ordinary business of the meeting having been discharged, the court became a special one for the alteration of certain clauses in the rules of the hospital.

